# Essential Genes of *Vibrio anguillarum* and Other *Vibrio* spp. Guide the Development of New Drugs and Vaccines

**DOI:** 10.3389/fmicb.2021.755801

**Published:** 2021-10-20

**Authors:** Michaël Bekaert, Nikki Goffin, Stuart McMillan, Andrew P. Desbois

**Affiliations:** Institute of Aquaculture, Faculty of Natural Sciences, University of Stirling, Stirling, United Kingdom

**Keywords:** antimicrobial resistance (AMR), aquaculture, transposon-insertion sequencing (Tn-seq), pathogen evolution, reverse vaccinology, *Vibrio cholerae*, *Vibrio parahaemolyticus*

## Abstract

Essential genes in bacterial pathogens are potential drug targets and vaccine candidates because disrupting their function is lethal. The development of new antibiotics, in addition to effective prevention measures such as vaccination, contributes to addressing the global problem of bacterial antibiotic resistance. The aim of this present study was to determine the essential genes of *Vibrio anguillarum*, a bacterial pathogen of aquatic animals, as a means to identify putative targets for novel drugs and to assist the prioritisation of potential vaccine candidates. Essential genes were characterised by a Tn-seq approach using the TnSC189 mariner transposon to construct a library of 52,662 insertion mutants. In total, 329 essential genes were identified, with 34.7% found within the core genome of this species; each of these genes represents a strong potential drug target. Seven essential gene products were predicted to reside in the cell membrane or be released extracellularly, thus serving as putative vaccine candidates. Comparison to essential gene data from five other studies of *Vibrio* species revealed 13 proteins to be conserved across the studies, while 25 genes were specific to *V. anguillarum* and not found to be essential in the other *Vibrio* spp. This study provides new information on the essential genes of *Vibrio* species and the methodology may be applied to other pathogens to guide the development of new drugs and vaccines, which will assist efforts to counter antibiotic resistance.

## 1. Introduction

Essential genes are those that when disrupted lead to the organism becoming non-viable because a critical biological process can no longer be accomplished (Jordan et al., 2002; Reznikoff and Winterberg, 2008), and this characteristic makes these genes and the products they encode important targets for the development of new antibiotics (Forsyth et al., 2002; Thanassi, 2002). The search for novel bacterial drug targets is now more urgent thanks to rising antibiotic resistance that renders many clinical classes of drugs potentially ineffective, with the situation particularly worrisome for Gram-negative pathogens (Aslam et al., 2018; Breijyeh et al., 2020; De Oliveira et al., 2020). Moreover, products of essential genes are strong candidates for vaccine development, particularly those encoding proteins that are released from the cell or expressed at the cell surface, as these may be recognised by the host to elicit long-term specific immune protection (García-Quintanilla et al., 2016b; Naz et al., 2019).

Essential genes are discovered through their inactivation, which may be achieved by targeted approaches such as transposon mutagenesis, CRISPR, and antisense RNA, though the most common approach is random transposon mutagenesis (van Opijnen and Camilli, 2013; Peters et al., 2016). Typically, this latter method requires conjugation of a suicide vector plasmid carrying a transposon from a donor bacterium into the recipient host, followed by transposon excision from the plasmid and insertion at a non-specific site in the host genome, thus disrupting the gene at that particular locus. Massively parallel sequencing approaches have greatly assisted the discovery of essential genes because the insertion sites of thousands of transposon-insertion mutants can be characterised in a single protocol (van Opijnen et al., 2009; Le Breton et al., 2015). The transposon-insertion sequencing (Tn-seq) method relies on a mariner-based transposon that inserts at TA dinucleotide sites, which occur approximately every 11–16 base pairs in bacterial genomes, meaning an excellent coverage of disrupted genes can be achieved (Judson and Mekalanos, 2000; Rubin et al., 2015).

Essential genes have been identified for human pathogens, such as *Bacillus subtilis* (Kobayashi et al., 2003; Peters et al., 2016), *Burkholderia pseudomallei* (Moule et al., 2014), *Escherichia coli* (Gerdes et al., 2003; Baba et al., 2006; Goodall et al., 2018; Martínez-Carranza et al., 2018), *Pseudomonas aeruginosa* (Gallagher et al., 2011; Turner et al., 2015; Poulsen et al., 2019), *Salmonella enterica* serovar *Typhimurium* (Barquist et al., 2013), *Shigella flexneri* (Freed et al., 2016), *Staphylococcus aureus* (Ji, 2001; Forsyth et al., 2002; Chaudhuri et al., 2009), and *Vibrio cholerae* (Cameron et al., 2008; Chao et al., 2013; Kamp et al., 2013); however, few studies concern bacteria species that infect fish, despite the problems caused by antibiotic-resistant pathogens in global aquaculture (Pham-Duc et al., 2019; Vincent et al., 2019; Reverter et al., 2020; FAO, 2021), which has led to a need for new therapies and vaccine candidates. Vaccination of fish has reduced antibiotic use considerably when farming certain species, such as Atlantic salmon (Sommerset et al., 2005), and this approach is suitable for other finfish. Still, most fish vaccines contain whole cells of inactivated pathogens and subunit vaccines, where only selected antigens are included in the preparation, could offer improved efficacy and safety profiles (Hansson et al., 2000; Adams, 2019; Ma et al., 2019).

*Vibrio anguillarum* is a fermentative, curved, Gram-negative bacterium responsible for vibriosis outbreaks in many species of fish, crustaceans and molluscs (Thakur et al., 2003; Toranzo et al., 2005; Frans et al., 2011; Marcos-López et al., 2013). Like other *Vibrio* spp., the *V. anguillarum* genome consists of two differently sized chromosomes, while plasmids may also be present such as the ca. 65-kDa pJM1 (and related plasmids) that encodes numerous virulence factors (Di Lorenzo et al., 2003; Okada et al., 2005; Naka et al., 2011). Vibriosis is a potentially lethal infection that is treated with antibiotics, but resistance can emerge and there are broader concerns for the impact of applying these agents in aquatic systems (Frans et al., 2011; Reverter et al., 2020; Desbois et al., 2021). Inactivated whole-cell vaccines are available to protect some species of fish against *V. anguillarum* (Colquhoun and Lillehaug, 2014), but these do not protect against every strain of the pathogen, and opportunities exist to develop more effective and safer subunit vaccines.

Essential genes in *Vibrio* spp. have been studied previously, and typically these have been consistent with work on many other free-living bacteria that show ca. 10–20% of genes to be essential for growth *in vitro* (Gerdes et al., 2003; Gil et al., 2004; Peters et al., 2016). Chao et al. (2013) identified 343 essential genes in *V. cholerae*, while Kamp et al. (2013) used a different isolate to identify 414 essential genes in this species. Cameron et al. (2008) determined 789 essential genes in *V. cholerae* but acknowledged that some of these were probably included by chance and Chao et al. (2013) provided some support to this suggestion. Chao et al. (2013) found most essential genes to be involved in “metabolism” and “translation,” but the functions of many other genes were uncharacterised. Hubbard et al. (2016) identified 565 essential genes in *V. parahaemolyticus*, and the functions of many of these genes were also undetermined. Still, there was a high degree of overlap in the essential gene sets in *V. parahaemolyticus* and the *V. cholerae* study of Chao et al. (2013), with 69% of the *V. parahaemolyticus* essential genes having homologs in the *V. cholerae* list (Hubbard et al., 2016). Guanhua et al. (2018) found 473 essential genes in *V. anguillarum* MVM425, many of which were hypothetical or of unknown function. Of note, these previous *Vibrio* spp. studies did not seek to identify gene products predicted to be released from the cell or expressed at the cell surface such that they could be developed as candidates for novel vaccines.

The aim of this present study was to determine the essential genes of *V. anguillarum* to identify putative targets for novel drugs and to assist the prioritisation of potential vaccine candidates for development and inclusion into a subunit vaccine. To achieve this, essential genes were identified by a Tn-seq approach, and then the subcellular locations of the products of each essential gene, their functions and metabolic pathways to which they contribute were determined. Finally, comparison was made to earlier studies of essential genes in *V. cholerae, V. parahaemolyticus* and *V. anguillarum* to shed light on the conservation of this set of fundamentally important genes across the genus and which guides the development of new antibacterial approaches against *Vibrio* spp.

## 2. Materials and Methods

### 2.1. Bacteria and Culture Media

*Vibrio anguillarum* NB10Sm, a spontaneous streptomycin [STR]-resistant strain of *V. anguillarum* NB10, a pathogenic isolate of the O1 serotype (Holm et al., 2015), was used as the recipient strain for transposon mutagenesis. The donor strain carrying the TnSC189 transposon on the pSC189 plasmid was *E. coli* SM10λpir (resistant to kanamycin [KAN]); pSC189 contains a resistance gene to ampicillin (AMP). TnSC189 had an *Mme*I restriction site introduced into the inverted repeat sequence at the 5′ end (van Opijnen and Camilli, 2013). Tryptone soy agar (TSA) and broth (TSB) and lysogeny agar (LBA) and broth (LBB) were prepared according to manufacturer instructions. To facilitate culture of *V. anguillarum*, the media were supplemented with 10–15 parts per thousand (ppt) sodium chloride (supplement in ppt given as superscript). Antibiotics were added to media as required (final concentration in mg/L given as superscript).

### 2.2. Transposon Mutagenesis and Extraction of Genomic DNA

Donor and recipient strains were incubated to early stationary phase (*E. coli*: LBB+AMP^100^, 37℃, 120 rpm, 4 h; *V. anguillarum*: TSA^15^ + STR^200^, 22℃, 150 rpm, 18 h), before combining at a CFU ratio of 1:1. Aliquots were pipetted onto 0.22-μm nitrocellulose filters on LBA^10^ plates and incubated (30℃, 24 h). Bacteria from the filters were collected in TSB^15^ and plated across TSA^15^+KAN^250^+STR^100^ and incubated (22℃, 72 h) to select for *V. anguillarum* containing the TnSC189 transposon. Colonies were collected, incubated (22℃, 150 rpm, 1 h), harvested by centrifugation and stored at −70℃ until required. This process was repeated to give three independent transposon insertion mutant libraries. Finally, duplicate 0.5-mL aliquots of each library were cultured in 5 mL TSB^15^ + KAN^250^ + STR^100^ (22℃, 150 rpm, 3 h) before genomic DNA from 3 mL of each of the six cultures was extracted according to Bartie et al. (2020).

### 2.3. Preparation of DNA for Sequencing

The genomic DNA was prepared for sequencing based on van Opijnen and Camilli (2013). The DNA was cleaved with *Mme*I that produces a staggered cut approximately 16-bp downstream of the transposon-located recognition site, thus within the genomic DNA of the bacterium, and which can be used to determine the insertion locus (van Opijnen and Camilli, 2013). The DNA was washed to remove protein, precipitated with sodium acetate, washed twice in 70% ethanol, and finally resuspended in water. Sequencing adaptors to allow amplification by PCR were ligated to the sticky ends resulting from digestion. DNA fragments (140 bp) across the junction of the transposon and genomic DNA were amplified by PCR following a modified version of the Illumina 16S Metagenomic DNA sequencing library preparation protocol (Illumina, 2016). Reaction products were purified by magnetic beads (Agencourt AMPure XP magnetic beads; Beckman Coulter UK Ltd, High Wycombe, UK). A second PCR attached the Illumina Nextera XT v2 index primers, with each sample amplified in duplicate and barcoded uniquely to distinguish them during the parallel sequencing of the libraries.

### 2.4. Sequencing of Libraries

Each of the 12 libraries were standardised to 20 nM in EB Buffer (Qiagen), pooled and adjusted to 10 nM. This master sample was diluted in HT1 buffer (Illumina) to 11 pM and PhiX (Illumina) was added to 20 pM to improve sequence read quality. The sample was sequenced on a MiSeq platform (Illumina) with a 50-cycle v2 reagent kit to generate up to 15 M single-end reads (Illumina).

### 2.5. Mapping of Transposon Insertion Locations

Reads from the sequenced libraries were filtered for quality (QC <25), length (50 nt) and complexity (entropy over 15) using fastp (Chen et al., 2018). Remaining reads were processed through cutadapt v2.10 (Martin, 2011) to remove the transposon sequences and Tn-seq primers/adaptors. Then, the genomic DNA sequences flanking the sites of transposon insertion were mapped to the *V. anguillarum* NB10 genome (Assembly GCF_000786425.1; NB10Sm has 107 fewer transposase genes than the published parent stain NB10) and plasmids using bowtie2 v2.3.5.1 (Langmead and Salzberg, 2012): Chromosomes I and II (NZ_LK021130.1 and NZ_LK021129.1, respectively) and 8 k (pL02, NC_009351.1), 6 k (pJV, NC_019325.1) and 67 k (p67vangNB10 virulence plasmid, NZ_LK021128.1) plasmids, allowing for no base mismatches. Reads mapping to multiple TA sites were distributed between the multiple possible sites (Hubbard et al., 2016). The number of unique insertion sites were counted, and their locations plotted with visual rendering generated by GView v1.7 (Petkau et al., 2010).

### 2.6. Identification of Essential Genes

A Python implementation of EL-ARTIST (Pritchard et al., 2014) was used to normalise the dataset for origin proximity and smooth the transposon insertion dataset using hidden Markov model analysis following sliding window training (50 bp; *P*-value > 0.005). Each gene was classified as “essential” (i.e., absence of an insertion in the gene sequence), “domain-essential” (i.e., insertions present only at the end of the sequence [continuously]) or “non-essential” (i.e., presence of insertions [dis-continuously or continuously in the totality of the sequence]). Sequence reads that failed to tally with a TA site in the *V. anguillarum* NB10 genome were kept only if coverage was higher than two reads, to account for divergence between the sequences of the reference and isolate used herein.

### 2.7. Predicted Functions and Subcellular Locations of Essential Genes

For genes identified as essential, work was performed to determine putative functions and subcellular locations in the bacterium. The annotated, hypothetical and pseudo-genes were functionally re-classified using InterProscan v5.44-79 (Jones et al., 2014; Mitchell et al., 2019) and KofamKOALA v95.0 (Aramaki et al., 2020). Protein subcellular locations were predicted using SignalP v5.0 (Almagro Armenteros et al., 2019), SecretomeP v2.0a (Bendtsen et al., 2005), and LipoP v1.0 (Juncker et al., 2003). The KEGG pathway enrichment analysis was done using bioconductor/DOSE v3.10 and R/clusterProfiler v3.14.3 (Yu et al., 2015) using the set of all *V. anguillarum* genes with KEGG annotation as reference. The Protein-protein interaction functional enrichment analysis was done using STRING v11.5 (Szklarczyk et al., 2021) and using the set of all *V. anguillarum* annotated genes as reference.

### 2.8. Comparison of Essential Gene Lists With *Vibrio* spp. Studies

Locus names/tags of essential genes from *V. cholerae* (Cameron et al., 2008; Chao et al., 2013; Kamp et al., 2013), *V. parahaemolyticus* (Hubbard et al., 2016) and *V. anguillarum* MVM425 (Guanhua et al., 2018) determined in previous studies were collected from the supplementary materials or by correspondence with the authors. These data were used to recover the nucleic acid sequence of each gene from the NCBI GenBank database. Sequence alignment using BlastN was performed to recover orthologous genes independent of the gene names because these can be misleading or incomplete. Gene sequences that had an alignment > 80% across > 80% of the gene length were considered to be orthologous. Subsequently, Venn diagrams to visualise the overlap in essential gene lists were generated by jVenn (Bardou et al., 2014).

### 2.9. Essential Genes in the Core Genome of *V. anguillarum*

A desirable drug or vaccine candidate would be conserved across the species, and so the essential genes identified in this present study were compared to the core genes (i.e., present in ≥ 95% of genomes) found in the 105 *V. anguillarum* genomes analysed by Coyle et al. (2020). PIRATE v1.0.4 (Bayliss et al., 2019) was used to build a comprehensive pangenome of *V. anguillarum*, and the essential genes identified using the Coyle et al. (2020) methodology. Analysis of the output was conducted using R v4.0.2 (R Core Team, 2020).

## 3. Results

### 3.1. Construction of Transposon Insertion Mutants

**Figure 1:**
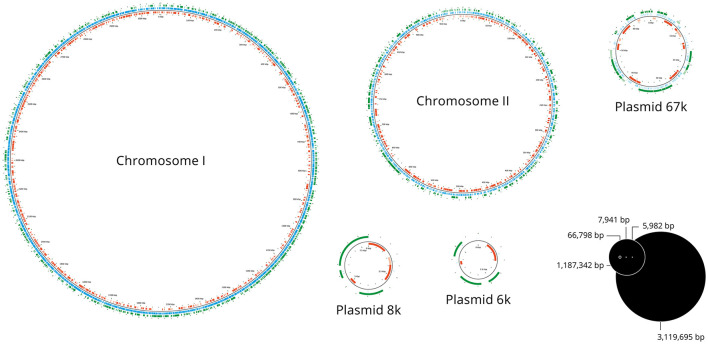
Map of transposon insertion sites. Representation of the two chromosomes and three plasmids of *V. anguillarum* NB10Sm showing the locations of the transposon insertions in the library of 52,662 mutants. The blue lines indicate the site of each transposon insertion, while green and red bars indicate the genes in forward and reverse orientations, respectively.

Three independent transposon insertion libraries were created in *V. anguillarum* NB10Sm using a mariner-based transposon that inserts at TA dinucleotide loci, and from these were collected an estimated 5,500, 9,500, and 15,300 mutant colonies, respectively; mean transformation efficiency was 1.10 × 10^-7^. The reads of three independent libraries that had been extracted and sequenced in duplicate were merged, giving a total of 5,802,645 reads. After filtering for sequence quality, length, and complexity, 4,727,608 reads (81.5%) were retained and aligned to the *V. anguillarum* NB10 genome and relevant plasmids (Supplementary Data 1). In total, 3,100,490 reads (65.6%) aligned exactly once to a complementary sequence in the reference genome, while 1,171,283 reads (24.8%) did not match any sequence; 455,835 reads (9.6%) aligned to more than one sequence and so were distributed evenly between the multiple possible sites (Figure 1). In total, 52,662 unique insertion locations were mapped from the three transposon insertion libraries, with the discrepancy in colonies vs. insertion locations most likely due to overlapping colonies not being counted separately.

### 3.2. Distribution of Transposon Insertion Sites

**Figure 2:**
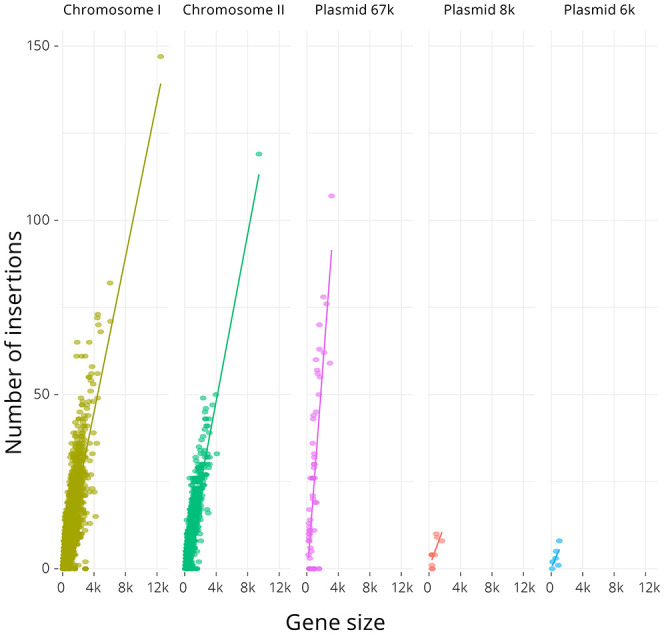
Relationship between gene sequence length (i.e., size) and number of transposon insertions for the two chromosomes and three plasmids in the *V. anguillarum* NB10Sm genome. There was significant correlation between gene sequence length and number of transposon insertions for the chromosomes and 67-kb plasmid and the *R*^2^ values were 0.61, 0.70, 0.60, 0.58, and 0.25 for Chromosome I, Chromosome II, Plasmid 67k, Plasmid 8k and Plasmid 6k, respectively. Slopes were calculated by regression where possible: 0.011, 0.012, and 0.029 for Chromosome I, Chromosome II, and 67-kb plasmid, respectively.

Chromosomes I and II have similar median gene lengths and were found to contain approximately the same abundance of insertions per gene (Table 1) but, despite having a median gene length similar to the chromosomes, the 67-kb pJM1-like virulence plasmid contained approximately twice as many transposon insertions per gene as the chromosomes (Table 1). As expected, there was strong correlation between gene length and number of insertions (Figure 2), with the chromosomes showing similar linear regressions (slopes of 0.011 and 0.012, respectively, ANOVA *P*-value = 0.69) that differed significantly from the regression of the 67-kb plasmid (slope of 0.029, ANOVA *P*-value <10^-15^). The linear regressions for the two smaller plasmids were not significant, due to the very low number of genes these encoded (5 and 10 genes). Across the genome, 20.7% of the TA insertion sites were disrupted by the transposon.

**Table 1:** Summary statistics of the *V. anguillarum* NB10Sm genome and transposon insertion mutant library.

	**Chromosome I**	**Chromosome II**	**Plasmid 67k**	**Plasmid 8k**	**Plasmid 6k**
Accession	NZ_LK021130.1	NZ_LK021129.1	NZ_LK021128.1	NC_009351.1	NC_019325.1
Median gene length (bp)	825	855	909	403.5	633
Median insertion number	9	9	16	4	3
Reads	2,124,734	678,730	296,905	69	52
TN inserts (possible)	177,722	71,101	4,483	531	594
TN inserts (targeted)	36,790	13,977	1,804	54	37
TN coverage (%)	20.70	19.66	40.24	10.17	6.23
Non-essential genes^*^	2,432	863	44	8	3
Essential genes^*^	259	62	4	2	2
Domain-essential genes^*^	68	26	1	0	0

*The genome was composed of two chromosomes and three plasmids*.

* *Post EL-ARTIST HMM analysis*.

### 3.3. Essential Genes

**Figure 3:**
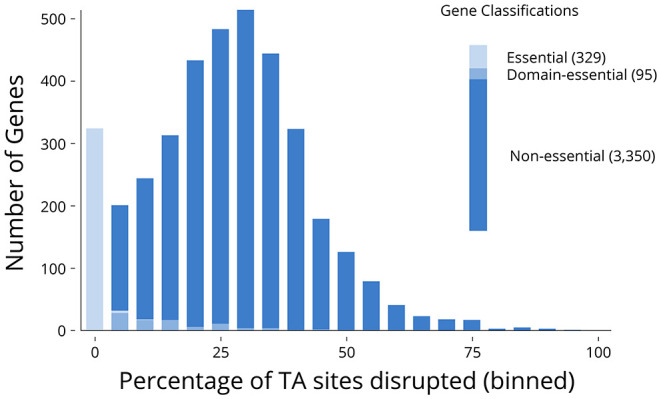
Classification of genes as essential, domain-essential, or non-essential. Distribution of percentage of TA sites disrupted in each gene (for those containing more than 10 TA sites in their sequence). Genes were categorised as essential, domain-essential, and non-essential within each bin (10-nt window) and in aggregate.

Of the 3,774 genes on the two chromosomes and three plasmids annotated in the *V. anguillarum* NB10Sm genome, 329 (8.7%) genes were classified as essential, with 95 (2.5%) classified as domain-essential (Figure 3; Supplementary Data 2). The essential genes were grouped together based on whether they encoded rRNA, tRNA, or protein. Notably, all 25 rRNA genes were classified as essential whilst, of the 93 tRNAs, 89 were classified as essential, with the remaining four classified as domain-essential. The genes encoding essential proteins were functionally re-annotated using the KEGG and InterPro databases (Table 2), which shed further light on their known or potential functions, including the pathways that had been disrupted to lethal consequence. Interestingly, one of the largest groups of essential genes with an ascribed function was those identified as “transposases,” which accounted for 40 of the 212 proteins (18.9%) in total.

**Table 2:** Classifications of the 329 essential and 95 domain essential genes found in *V. anguillarum* NB10Sm.

**Gene class**	**Essential present**	**Domain essential**
tRNA (out of 93)	89	4
rRNA (out of 25)	25	0
ncRNA (out of 1)	1	0
tmRNA (out of 1)	1	0
SRP RNA (out of 1)	1	0
Protein coding gene (out of 3,660)	212	91
Transposase	40	0
Ribosomal protein	33	11
Sulfur relay	16	6
Other	105	51
Uncharacterised function	18	23

*Genes were grouped together based on annotation and product description. Gene details are available in Supplementary Data S2. tRNA, transfer RNA; rRNA, ribosomal RNA; ncRNA, non-coding RNA; tmRNA, transfer-messenger RNA; SRP RNA, signal recognition particle RNA*.

### 3.4. Enrichment Analysis

Two KEGG pathways showed significant enrichment for essential genes (i.e., *P*-value <0.001). First is the “Ribosome” pathway (ko03010, Supplementary Figure 1A), where most of the 33 essential gene proteins contribute to the structure of the ribosome, in addition to the tRNA, rRNA, and the small number of elongation factors that are involved (adjusted *P*-value = 10^-23^); second is the “Sulfur relay system” pathway (ko04122, Supplementary Figure 1B), where 16 essential genes assist in the sulfur transfer steps of tRNA thiolation, folate biosynthesis, and thiamine and cysteine metabolism (adjusted *P*-value = 10^-6^). Similarly, STRING protein-protein interaction analysis revealed that “Ribosome and Protein biosynthesis,” where most of the 33 essential gene proteins contributed, was the most significant cluster (PPI enrichment *P*-value <10^−16^; expected interactions: 583; detected interactions: 2,869).

### 3.5. Core Genes

**Figure 4:**
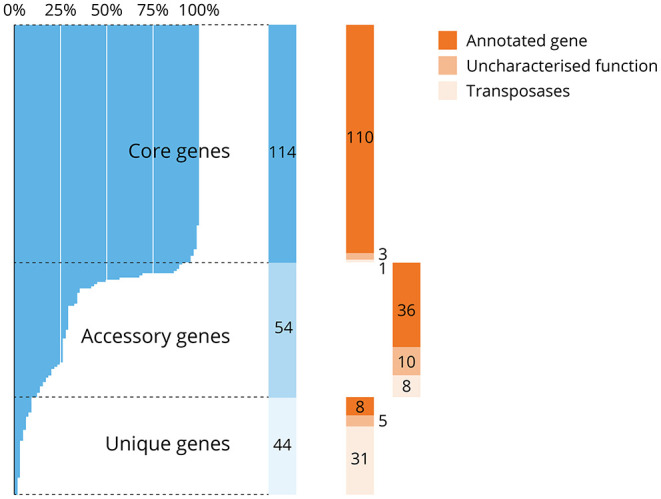
Distribution of protein-encoding essential genes in the pan-genome of *V. anguillarum*. Most essential protein-encoding genes (114/212, 53.8%) were found within the core genome of *V. anguillarum*, i.e., found in over 95% of the 105 genomes analysed, whilst 54/212 (25.5%) were in the accessory genome or detected uniquely in this isolate (44/212, 20.8%; in blue). Each essential gene was further characterised to be annotated, of or a transposase or a transposase (in orange). Most of the core genes were annotated (110/114, 96.5%) whilst most of the transposases were found uniquely in this isolate (31/44, 70.5%). Note that some core genes may have been incorrectly assigned to the accessory gene list due to available genomes being incomplete or of poor quality.

To assess the conservation of protein-encoding essential genes across the species *V. anguillarum*, the presence of the essential genes in the core genome of 105 sequenced isolates available at time of analysis was assessed according to the approach of Coyle et al. (2020). In total, 114 of the essential protein-encoding genes (53.8%) and 68 of the domain-essential genes (74.7%) were found within the core genome of *V. anguillarum*, and most of these core genes were annotated (110/114), with one transposase and only three genes of uncharacterised function (Figure 4). Fifty-four essential protein-encoding genes were within the accessory genome (i.e., found in at least 5% of genomes but <95% of genomes overall), which included ten uncharacterised proteins and eight transposase genes. Some of these essential genes determined to be part of the accessory genome may in fact be core genes, with the incompleteness of some published genomes meaning these genes are not found within a sufficient proportion of genomes to be classified as core. Of note, 44/212 (20.8%) of the essential protein-encoding genes were classified as unique (i.e., present in <5% of *V. anguillarum* genomes), whilst only eight essential genes with an annotation were unique.

### 3.6. Comparison of Essential Gene Sets in *Vibrio* spp.

**Figure 5:**
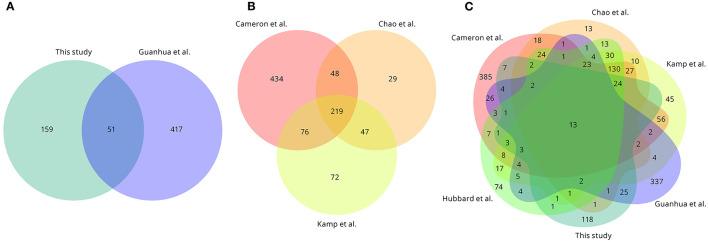
Conservation of essential protein-encoding genes across published *Vibrio* spp. studies. **(A)** Overlap of essential genes between *V. anguillarum* NB10Sm (this study) and *V. anguillarum* MVM425 (Guanhua et al., 2018). **(B)** Overlap of essential genes between three *V. cholerae* studies (Cameron et al., 2008; Chao et al., 2013; Kamp et al., 2013). **(C)** Overlap of essential genes between the six *Vibrio* studies. Note that for *V. parahaemolyticus* (Hubbard et al., 2016) only 402 genes out of 418 described were retrieved, as 16 loci were absent (e.g., VP0254). Duplicated genes are counted only once.

The essential gene list identified in this present study was compared with the lists determined in other *Vibrio* species and isolates (Figure 5; Supplementary Data 3). In total, 51 essential protein-encoding genes were shared between the two *V. anguillarum* isolates analysed (Figure 5A; Supplementary Data 3), and 36 (70.6%) of these genes were present within the core genome of this species. Comparison of the three studies to date on *V. cholerae* revealed 219 essential genes to be shared across these studies, with a further 171 genes essential in two of the three studies (Figure 5B; Supplementary Data 3). Taking together the three studies of *V. cholerae*, two studies of *V. anguillarum* and a single study of *V. parahaemolyticus*, there were 13 essential genes shared between the six studies, with nine of these genes encoding components of the 30S and 50S ribosomes and the other four genes described in the *V. anguillarum* NB10 genome to be a single-stranded DNA-binding protein; dihydropteroate synthase; co-chaperone *HscB*; and UDP-3-O-acyl-N-acetylglucosamine deacetylase (Figure 5C; Supplementary Data 3). Twenty-five essential genes were found exclusively in the *V. anguillarum* studies and were not present in the other *Vibrio* studies (Figure 5C; Supplementary Data 3). Finally, 118 protein-encoding genes in *V. anguillarum* NB10Sm were essential only in this isolate, though some of these genes are not reported to be present in the genomes of the isolates used in the other *Vibrio* studies (Figure 5C; Supplementary Data 3).

### 3.7. Subcellular Locations

Essential genes are potential drug targets, while extracellular products of essential genes and those expressed at the cell membrane would be strong candidates for immunogenicity assessment and consideration for possible inclusion into a vaccine. Hence, SecretomeP, SignalP, and LipoP were used to predict the subcellular locations of the essential gene products from *V. anguillarum* NB10Sm by identifying those that were non-classically secreted or contained a suitable signal peptide (Table 3). In total, seven essential gene products were predicted to be located to the inner cell membrane or the periplasm, including five genes found within the core genome of *V. anguillarum* (Table 3). Interestingly, two of these core genes (the SoxR reducing system protein gene *rseC* and the thiosulfate sulfurtransferase gene *pspE*), in addition to *bcrA* (benzoyl-CoA reductase subunit A gene) found in the accessory genome of *V. anguillarum*, were also essential in the other study performed on *V. anguillarum* MVM425.

**Table 3:** Essential genes encoding products predicted to be released from the cell or expressed at the cell surface.

**Gene ID^†^**	**Pan-genome**	**Shared**	**Annotation**	**Cell location**
RS10405	Core	1	*rsxB*—Electron transport complex subunit	Cell inner membrane
RS16490	Core	0	*cdsA*—Phosphatidate cytidylyltransferase	Cell inner membrane
RS17415	Core	2*	*rseC*—SoxR reducing system protein	Cell inner membrane
RS00530	Core	1*	*pspE*—Thiosulfate sulfurtransferase	Periplasm
RS02940	Unique	0	*Putative excinuclease*	Cell inner membrane
RS08840	Accessory	1*	*bcrA*—benzoyl-CoA reductase subunit A	Cell inner membrane
RS17130	Core	4	*murG* ^‡^	Cell inner membrane

Seven essential genes of V. anguillarum NB10Sm contained a significant peptide-signal and secretory pathway signal (P-value <10^-3^), with predicted subcellular locations as reported for E. coli. The seven genes were classified according to the pan-genome (see Figure 4) and were investigated for conservation within the six Vibrio essential gene studies (“Shared” column; see Figure 5), including three genes found to be shared with the V. anguillarum essential gene study of Guanhua et al. (2018) (*).

† *GeneID: VANGNB10_RSxxxxx*.

‡ *UDP-N-acetylglucosamine-N-acetylmuramyl-(pentapeptide) pyrophosphoryl-undecaprenol N-acetylglucosamine transferase*.

## 4. Discussion

In this present study, a Tn-seq approach to disrupting gene functions led to the determination of 329 essential and 95 domain-essential genes in the NB10Sm isolate of *V. anguillarum*, an aquatic pathogen that can infect many cultured aquatic species. Each of these essential genes is a potential target for the design of new chemotherapeutants, while subcellular location predictions *in silico* allowed for the generation of a shortlist of seven putative vaccine candidates containing proteins released from the cell or expressed at the cell surface. Comparison with studies of essential genes in another isolate of *V. anguillarum* and other *Vibrio* spp. allowed for the compilation of conserved lists of genes for prioritisation of the most promising antibacterial targets, whilst also advancing prospects for the development of genus- and species-specific antibacterial agents. Though essential genes have been identified in various human pathogens, including *Vibrio* spp., few studies have applied such an approach to fish pathogenic bacteria, and previous studies have not used these data to select possible *Vibrio* spp. vaccine candidates.

In total, 8.7% of the genes in the *V. anguillarum* NB10Sm genome were essential, with a further 2.5% classified as domain-essential, which is consistent with studies of other free-living bacteria that report typically 10–20% of total genes in a genome to be essential during culture in a rich medium *in vitro* (Gerdes et al., 2003; Gil et al., 2004; Peters et al., 2016). Other essential gene studies with *Vibrio* spp. employing various methods reported 343, 414, and 789 essential genes in *V. cholerae*, representing 9.9, 10.7, and 20.3%, respectively (Cameron et al., 2008; Chao et al., 2013; Kamp et al., 2013); 565 (12.7%) in *V. parahaemolyticus* (Hubbard et al., 2016); and 473 (12.5%) in *V. anguillarum* MVM425 (Guanhua et al., 2018).

Many of the essential protein-encoding genes in *V. anguillarum* NB10Sm were found within two pathways that contribute critically to protein synthesis, namely the “Ribosome” and “Sulfur relay system” pathways (ko03010 and ko04122, respectively), which is consistent with other studies that have found disruption of protein synthesis to be lethal in bacteria (Kobayashi et al., 2003; Chao et al., 2013; Hubbard et al., 2016). Indeed, several antibiotic groups disrupt ribosome function to lethal effect, including aminoglycosides and tetracyclines that target the 30S subunit (Fourmy et al., 1996; Chopra and Roberts, 2001) and the lincosamides and macrolides that bind to the 50S subunit (Spížek and Řezanka, 2017; Vázquez-Laslop and Mankin, 2018). The ribosome is the organelle where proteins are translated, meaning disruption of this key cellular machinery has a global impact on the cell's ability to synthesise proteins faithfully for normal cell functioning. All the tRNA genes were essential or domain-essential, which is to be expected given their vital role in protein synthesis, an observation with precedent (Reznikoff and Winterberg, 2008; Guanhua et al., 2018). Often tRNAs are excluded from essential gene lists due to their short sequence length (typically 76–90 bp in length), meaning there may not be sufficient confidence that the genes were not targeted by an insertion in Tn-seq libraries of lower saturation (Chao et al., 2016). However, in this present study, when the tRNA genes were considered as a concatenated sequence, the lack of transposon insertions was highly significant (*P*-value = 10^−21^), thus supporting their essentiality. Proteins in the sulfur relay system play crucial roles in tRNA thiolation, folate synthesis, and thiamine, cysteine and methionine metabolism, which are processes contributing to protein synthesis. Furthermore, rRNA composes the integral structure of the ribosome and all 25 rRNA genes in *V. anguillarum* NB10Sm were essential. This observation for the rRNA genes was made despite their redundancy, and this may be due to the mechanism of gene reversion that acts to maintain consistency of the genetic sequence at each locus (Liao, 2000; Santoya and Romero, 2005). As a result, the transposon-insertion sequence may have been either eliminated from each of the rRNA genes or propagated to each locus to likely lethal effect. Of course, the Tn-seq methodology only identifies the insertion locations in surviving bacteria, which would be those where the insertion had been eliminated, thus the genes would appear to lack insertions and be classified as essential. The high number of transposase or transposase-like genes classified as essential, particularly those belonging to the IS66 family, were mostly only found in the isolate used in this present study (31/40; 77.5%). The consistent lack of insertions into such genes may be due to the key roles they have in maintaining genome structure (Vigil-Stenman et al., 2017), but it may also be that these sequences possess mechanisms that protect against insertion. Finally, many essential genes were of uncharacterised function, which is familiar for studies of this nature (Hubbard et al., 2016), and it serves to draw attention to the shortcomings in our understanding of fundamental aspects of the biology of many genes in bacterial genomes.

Amongst the 13 essential genes conserved across each of the six studies of *Vibrio* spp. was the gene encoding UDP-3-O-[3-hydroxymyristoyl] N-acetylglucosamine deacetylase, *LpxC*. LpxC is a key enzyme in the biosynthesis of Lipid A, a key structural component of the Gram-negative outer membrane, where it acts to anchor the lipopolysaccharide (Emiola et al., 2015; Joo, 2015). Compounds targeting this enzyme have been sought previously because it is conserved across Gram-negative species (Langklotz et al., 2011; Titecat et al., 2016) and its inactivation represents a novel means to combat bacterial pathogens (Williams et al., 2006; Tomaras et al., 2014; García-Quintanilla et al., 2016a). In addition to studies of various bacteria where *LpxC* is essential (Akerley et al., 2002; Barquist et al., 2013), the finding that this gene was essential in all six *Vibrio* spp. studies, confirms its value as a putative drug target and provides further support for the bioinformatics-driven approach to drug target prioritisation demonstrated herein.

Twenty-five essential genes were found exclusively in the *V. anguillarum* studies and were not present in the other studies of *Vibrio* spp., which could be exploited to develop more specific-acting antibacterials. This may be desirable because many non-pathogenic *Vibrio* spp. are important to the normal growth, development and health of the farmed aquatic animals, and they are often found within the animals' microbiota and in the culture water (Gajardo et al., 2016; Bone et al., 2021; Lorgen-Ritchie et al., 2021). Essential genes within the core genome of a bacterial species will be of greater interest for drug and vaccine development, particularly if they are essential across multiple isolates, because this allows for the effective targeting of most strains of a pathogen. A shift away from application of broad-spectrum antibiotics to agents with a narrow spectrum that target only the intended bacterium is one approach to reducing the problem of bacterial antibiotic resistance, as this avoids applying selection pressure for resistance on non-target species (Melander et al., 2018).

This present study demonstrated a new approach to identifying and prioritising potential candidates for incorporation into subunit vaccines for *Vibrio* spp. by applying bioinformatics tools to identify proteins of essential genes predicted to be located in the cell membrane or released extracellularly. All seven of the proteins identified by this approach in *V. anguillarum* NB10Sm, including three identified to be essential in *V. anguillarum* MVM425 (Guanhua et al., 2018), were predicted to be located at the inner cell membrane or in the periplasm, perhaps suggesting they may not be as detectable by host immune cells where the adaptive response is ideally desired. None of these seven genes were found amongst the 13 essential genes conserved across the six *Vibrio* spp. studies (Figure 5). Although, follow-up work would need to assess the immunogenicity of the surface-expressed or extracellular proteins, this present study does demonstrate the potential usefulness of such an approach.

Importantly, the differences between the essential gene lists derived from two phylogenetically closely related isolates, i.e., *V. anguillarum* NB10Sm and MVM425 (Coyle et al., 2020), and determined by a similar approach, demonstrates the value of characterising essential genes in multiple isolates of the same species when seeking conserved targets (Martínez-Carranza et al., 2018). An unexplained distinction between the *V. anguillarum* studies was the presence of 91% of the essential genes in MVM425 to be located on Chromosome I (Guanhua et al., 2018) despite comprising 72% of the genome, which compared to 79% of essential genes on Chromosome I in this present study where this chromosome accounted for 73% of the genome. Collectively, inconsistencies between similar studies exposes our still primitive understanding of essential genes, including their roles in the host and “rules” underpinning essentiality, further justifies their continued investigation.

The approach described in this present study is limited by the application of different approaches to identifying and classifying essential genes between studies. Even so, such distinctions between experiments allow for further rationalisation of conserved gene lists and provide stronger support for targeting the genes ultimately identified. Unquestionably, the use of pharmacological agents with specific action against the products of the essential genes, or the generation of targeted mutants, would have strengthened the case for essentiality and such approaches will be necessary to confirm this trait for each candidate essential gene (Falconer et al., 2011; Fields et al., 2017). Likewise, the essentiality of the genes has been determined *in vitro* only and investigation of their importance in more natural conditions, such as in seawater or during an infection, are likely to uncover slightly different gene lists, as these depend entirely on the conditions in which they are generated (Freed et al., 2016; Fields et al., 2017). Nevertheless, the combination of transposon-based gene disruption and high-throughput sequencing technologies allowing for rapid mapping of insertion locations across entire genomes is revolutionising our understanding of the essentiality of genes in bacterial genomes, whilst assisting to uncover gene functions through experiments under selective conditions (van Opijnen and Camilli, 2013; Chao et al., 2016). This present study adds to the increasing number of reports on essential genes in bacteria, which provide fundamental insights into genetic and metabolic networks that can also inform the creation of synthetic microorganisms with minimised genomes, thereby helping to unlock their exciting potential (Gil et al., 2004; Hutchison et al., 2016).

In conclusion, the approach demonstrated here provides a means to find new vaccine candidates and bacterial targets for the development of novel antibiotics, including agents of varying specificity. The methodology can be applied to different pathogens to guide the discovery of new measures to combat infectious diseases. Multiple approaches, including the discovery of new antibacterial agents and effective vaccination, are needed to address the global issue of antibiotic resistance, a classical One Health problem with human, animal, and environmental components.

## Data Availability Statement

The Tn-seq data were submitted to the EBI ENA database under project number PRJEB39186. The scripts and pipeline used to process the Tn-seq reads are available at https://github.com/pseudogene/vibrio-tnseq.

## Ethics Statement

This study was performed in accordance with the ethical review procedures of the University of Stirling.

## Author Contributions

AD and SM conceived and designed research and conducted the experiments. MB contributed new analytical tools. NG, MB, and AD analysed the data. MB and AD wrote the manuscript. All authors read and approved the manuscript.

## Funding

SM was funded in part by a University of Stirling Collaborative Research Studentship with the Centre for Environment, Fisheries and Aquaculture Science.

## Conflict of Interest

The authors declare that the research was conducted in the absence of any commercial or financial relationships that could be construed as a potential conflict of interest.

## Publisher's Note

All claims expressed in this article are solely those of the authors and do not necessarily represent those of their affiliated organizations, or those of the publisher, the editors and the reviewers. Any product that may be evaluated in this article, or claim that may be made by its manufacturer, is not guaranteed or endorsed by the publisher.
