# Supplementary material for: Essential Genes of Vibrio anguillarum and Other Vibrio spp. Guide the Development of New Drugs and Vaccines
Source: Front Microbiol. 2021 Oct 20;12:755801. doi: 10.3389/fmicb.2021.755801 (PMC8564382; doi:10.3389/fmicb.2021.755801)
Supplement: Supplementary file 1 [file Data_Sheet_1.PDF]

Essential genes of *Vibrio anguillarum* and other *Vibrio* spp. guide the development of new drugs and vaccines

SUPPLEMENTARY FIGURE

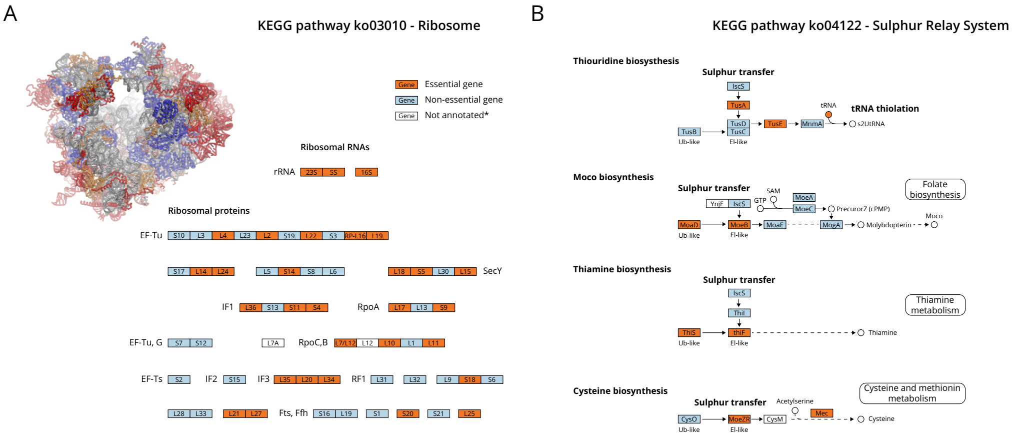

Supplementary Figure S1. KEGG pathways of significance following the enrichment analysis. **(A)** KEGG pathway "Ribosome", **(B)** KEGG pathway "Sulfur relay system". Not annotated*: Gene not present in the current genome annotation.

SUPPLEMENTARY DATA

Supplementary Data S1

Detailed insertions locations and numbers in each chromosome and plasmids. (CSV)

Supplementary Data S2

Details of the essential genes. For each of the 329 genes, the genomic location, functional re-annotation and sequences are provided. (CSV)

Supplementary Data S3

Shared genes between studies. All *Vibrio* spp. studies together, *V. anguillarum* studies only, as well as the *V. anguillarum* specific genes. (CSV)
